# Epidemiological changes in *Chlamydia pneumoniae* molecular detections before, during and after the COVID-19 pandemic in 27 European sites and Taiwan, 2018 to 2023

**DOI:** 10.2807/1560-7917.ES.2025.30.23.2400682

**Published:** 2025-06-12

**Authors:** Florian Tagini, Søren Anker Uldum, Carla Berengua, Branislav Ivan, Riccarda Capaul, Sophie Edouard, Adrien Fischer, Jacky Flipse, Diego García Martínez de Artola, Daniel Goldenberger, Edou Heddema, Mirjam Hermans, Frank Imkamp, Darja Keše, Clara Lejarraga, Reto Lienhard, Carola Maffioli, Veerle Matheeussen, Patrick M Meyer Sauteur, Irena Mitrovic, Onya Opota, Christina Orasch, Pavel Drevinek, Olivia Peuchant, Liu Po-Yu, Mirja Puolakkainen, Melissa Remy, Khoa TD Thai, Nadia Wohlwend, Gilbert Greub, on behalf of the ESCMID Study Group for Mycoplasma and Chlamydia infections (ESGMAC)

**Affiliations:** 1 Institute of Microbiology, Lausanne University Hospital, Lausanne, Switzerland; 2 Department of Bacteria, Parasites and Fungi, Statens Serum Institut, Copenhagen, Denmark; 3 Microbiology Department, Hospital de la Santa Creu i Sant Pau, Barcelona, Spain; 4 University Hospital Basel, Basel, Switzerland; 5 Zentrallabor, Kantonspital Graubünden, Chur, Switzerland; 6 IHU-Méditerranée Infection, Marseille, France; 7 Bacteriology Laboratory, Division of Laboratory Medicine, Department of Diagnostics, Geneva University Hospitals, Geneva, Switzerland; 8 Laboratory for Medical Microbiology and Immunology, Rijnstate Hospital, Arnhem, the Netherlands; 9 Hospital Universitario Nuestra Señora de Candelaria, Santa Cruz de Tenerife, Spain; 10 Zuyderland Medical Centre, Glims, Sittard-Geleen, the Netherlands; 11 Jeroen Bosch Hospital, s-Hertogenbosch, the Netherlands; 12 University of Zurich, Institute of Medical Microbiology, Zürich, Switzerland; 13 Laboratory for diagnostics of atypical bacterial infections, Institute of Microbiology and Immunology, Faculty of Medicine, Ljubljana, Slovenia; 14 Microbiology laboratory, HUC, Bilbao, Spain; 15 ADMED Microbiologie, La Chaux-de-Fonds, Switzerland; 16 MCL Laboratories, Niederwangen, Switzerland; 17 University Hospital Antwerp, Antwerp, Belgium; 18 Infectious Diseases and Hospital Epidemiology, University Children's Hospital Zurich, Zurich, Switzerland; 19 Luzerner Kantonsspital, Lucerne, Switzerland; 20 MEDISYN SA, Lucerne, Switzerland; 21 Motol University Hospital, Prague, Czechia; 22 Department of Bacteriology, Bordeaux University Hospital, Bordeaux, France; 23 Taichung Veterans General Hospital, Taichung, Taiwan; 24 University of Helsinki and Helsinki University Hospital, Helsinki, Finland; 25 Star-shl, Rotterdam, the Netherlands; 26 Laboratory Dr. Risch, Buchs, Switzerland; 27 https://www.escmid.org/esgmac/

**Keywords:** pneumonia, bronchitis, asthma, outbreak, upsurge, PCR, molecular diagnosis

## Abstract

**Background:**

During the COVID-19 pandemic, non-pharmaceutical interventions (NPIs) such as social distancing, lockdowns and enhanced hygiene led to a decrease in respiratory pathogens. However, as NPIs were relaxed, a resurgence in several respiratory pathogens was observed including one local *Chlamydia pneumoniae* outbreak in Switzerland, prompting the need for a better understanding of *C. pneumoniae* epidemiology.

**Aim:**

To assess temporal and geographical variations in *C. pneumoniae* detection before, during and after the COVID-19 pandemic.

**Methods:**

Data on *C. pneumoniae* PCR detection ratios (number of positive tests/ total number of tests) across pre-pandemic (2018–2019), pandemic (2020–2022) and post-pandemic (2023) periods were collected via a global survey disseminated through various professional networks.

**Results:**

*C. pneumoniae* detection ratios were analysed across 28 sites (27 in Europe, one in Taiwan) in 2023 (Dataset A, n = 172,223 tests) and 20 sites from 2018 to 2023 (Dataset B, n = 693,106 tests). Twenty-seven sites were laboratories (hospital or clinical) and one a surveillance system (Denmark). A significant decrease in detection ratios was observed during the pandemic period (from 1.05% to 0.23%, p < 0.001). In 2023, detection ratios increased to 0.28% (p < 0.002). Notable regional variations were found, with statistically significant increases in detection ratios at six sites located in Switzerland and Slovenia, where ratios ranged from 0.52% to 3.25%.

**Discussion:**

The study highlights how NPIs influenced *C. pneumoniae* epidemiology, with reduced detection during the pandemic and partial resurgence afterwards. Regional variations suggest differing NPI impacts and underscore the need for continued surveillance.

**Key public health message**

**What did you want to address in this study and why?**

*Chlamydia pneumoniae* is a bacterium that causes respiratory tract infections. Due to observed shifts in patterns of some respiratory pathogens following the lifting of non-pharmaceutical interventions (NPIs), such as lockdowns and social distancing, after the COVID-19 pandemic, we wanted to observe patterns of *C. pneumoniae*. Furthermore, a local outbreak in Switzerland in late 2023 raised concerns about a potential global resurgence.

**What have we learnt from this study?**

We found that *C. pneumoniae* detection was reduced during the pandemic period (2020–2022) when NPIs were in place and partially rebounded in 2023, although with a delay, after most NPIs were lifted. Regional variations in resurgence patterns suggest differing impacts of NPIs on pathogen transmission and indicate the potential for future resurgences.

**What are the implications of your findings for public health?**

Ongoing surveillance is crucial for understanding the long-term epidemiological trends of *C. pneumoniae* and preparing for potential resurgences in countries that have not experienced any increase in detection rates.

## Introduction

During the first 2 years of the COVID-19 pandemic (2020–2022), the incidence of many pathogens, including respiratory pathogens such as influenza viruses, respiratory syncytial virus (RSV) and *Mycoplasma pneumoniae*, decreased. This was due to the implementation of non-pharmaceutical interventions (NPIs) such as social distancing, lockdowns, school closures, stay-at-home orders, mask-wearing and enhanced hygiene practices, including widespread use of hydroalcoholic solutions for hand hygiene [1-4]. However, as these NPIs were lifted, a global resurgence of these pathogens was observed, with some, such as *M. pneumoniae* and *Streptococcus pyogenes*, showing infection rates even higher than those seen before the COVID-19 pandemic [5-7]. This resurgence was likely due to decreased levels of specific immunity against airborne pathogens.

*Chlamydia pneumoniae* is an intracellular bacterium responsible for respiratory infection ranging from upper respiratory tract infection to bronchitis and pneumonia [8]. In general, pneumonia is typically mild with a favourable prognosis and low mortality rate (< 2%) [9,10]. However, severe cases may occasionally be observed resulting in hospitalisation, sporadically accompanied by complications such as pleural effusion or empyema, and the need for intensive care [9,11,12]. Seroepidemiological studies indicate that primary infections are rare in children under 5 years old in temperate regions [13]. When primary infection occurs in adults, it can lead to more severe clinical outcomes compared with reinfection [11]. There is a strong association between *C. pneumoniae* and asthma initiation, persistence, exacerbation and treatment resistance [14,15]. *C. pneumoniae* infections can be treated with either macrolides, fluoroquinolones or doxycycline [16]. In recent years, molecular diagnosis has emerged as the best technique to diagnose acute infections caused by this intracellular bacterium, showing excellent sensitivity and specificity [17-19]. The pathogen DNA can be found in abundance in respiratory samples such as nasopharyngeal swabs, sputa or bronchial aspirates and broncho-alveolar lavages. Multiplexing has also proven to be efficient, as coinfections frequently occur (and as bystander, allows the monitoring of epidemiological trends of various respiratory pathogens) [20-22]. Serology demonstrates lower sensitivity than PCR testing, particularly if the sample is taken during the serological window (when patients are infected but have not yet produced detectable levels of antibodies) or if convalescent serum is unavailable. It also demonstrates lower specificity due to cross-reactivity and the influence of past infections, and considerable variability between different serological assays has been reported compared with PCR [23,24]. In addition, cell culture is not suitable for routine diagnosis.

In the past several years, a few *C. pneumoniae* outbreaks have been reported, such as in a prison in Texas, United States in 2013 (52 cases identified) and in South Korea in 2016 where 19 cases were reported in children [10,25]. From our literature search, we did not find reports of outbreaks occurring between 2016 and 2022. To our knowledge, the most recent outbreak to be reported occurred in Lausanne, Switzerland in late 2023 after the COVID-19 pandemic [26]. Between October and December 2023, routine epidemiologic surveillance at Lausanne University Hospital in Switzerland revealed an unexpected rise in *C. pneumoniae* PCR detection ratios, reaching 3.61% overall and peaking at 6.66% in October, with a total of 28 patients identified. These figures contrast with the hospital’s historical rates over the past decade, which typically ranged from 0% to 0.75%. This unexpected surge prompted us to conduct a multicentric epidemiological study on *C. pneumoniae* infections to determine whether similar patterns are emerging globally, particularly in the context of the observed resurgence of other pathogens following the COVID-19 pandemic. Thus, we, through the European Society of Clinical Microbiology and Infectious Diseases (ESCMID) Study Group for Mycoplasma and Chlamydia Infections (ESGMAC), performed a survey aiming to assess temporal and geographical variations in *C. pneumoniae* detection before, during and after the COVID-19 pandemic.

## Methods

### Survey development and dissemination

A structured survey was adapted from the survey developed for the study of *M. pneumoniae* detection ratios following established guidelines for survey research by Patrick Meyer-Sauteur et al. [2]. It was adapted to collect data on *C. pneumoniae* detection ratios. The survey included four sections: (i) participant details, (ii) information about the testing laboratory and region, (iii) detailed information on the testing method for *C. pneumoniae* detection (including technique, product and manufacturer or reference), (iv) *C. pneumoniae* molecular testing data (total tests, positive tests, positive tests by month, proportion of children/adolescents under 18 years and females of any age). The survey was conducted in English and hosted on the SurveyMonkey online platform (SurveyMonkey Inc, San Mateo, California, US, www.surveymonkey.com). Additional details of the survey are provided in the Supplementary material.

The survey was disseminated worldwide to hospital laboratories, infectious disease specialists and members of national surveillance systems (with contacts in Europe more represented) through various channels, including ESCMID study groups (ESGMAC, the ESCMID Study Group for Genomics and Molecular Diagnostics (ESGMD), the ESCMID Study Group for Epidemiological Markers (ESGEM)) and national infectious diseases and microbiology societies via newsletters or email distribution lists, social media (Swiss Society for Microbiology) and direct in-person contact by some authors (FT and GG). The survey was advertised during an ESGMAC webinar in January 2024. Potential participants were contacted and provided with a summary of the study and a link to the online survey. Participation was on a voluntary basis and without any financial compensation. Consent for data publication and participant listing was obtained on the first page of the questionnaire. The survey was launched on 1 December 2023, with reminders sent to potential participants via email after 6 and 10 weeks. We closed the survey on 31 March 2024. Each entry, originating from a laboratory (hospital or clinical) or a surveillance system was considered a site.

### Data quality control

Each entry was carefully checked for (i) data completeness, (ii) data origin, and (iii) molecular diagnostics method. Furthermore, we assessed each entry to prevent data duplication by thoroughly checking for similarities in the introduced numbers.

### Case definition

We included detection ratios from PCR tests only, due to improved sensitivity and specificity of PCR compared to serology. Each positive PCR result was considered a potential case. We analysed PCR detection ratios (number of positive tests divided by the total number of tests over a defined period) in this study, not incidence or prevalence as we could not control for multiple testing of the same individual.

### Statistical analysis

To analyse the results, three periods were defined: 2018–2019 (the situation before the COVID-19 pandemic, also referred as the pre-pandemic period), 2020–2022 (the situation during COVID-19 pandemic, the pandemic period) and 2023 (after the COVID-19 pandemic, also referred as the post-pandemic period). The proportion of children/adolescents and females were calculated as follows: number of tests performed on children/adolescents younger than 18 years old, or in females, respectively, divided by the total number of tests performed. For global comparison, detection ratios between the periods were analysed using the Wilcoxon signed rank test with continuity correction (data were indicated as paired). Significance was set to p < 0.05. Then, the three periods were analysed pairwise using contingency tables with Fisher’s exact test in R software version 4.2.2 [27]. Detection ratios were analysed as no denominator was available to calculate incidence rates. P value significance level was adapted according to Benjamini-Hochberg correction for multiple testing [28].

## Results

### Study population and detection methods

**Figure 1:**
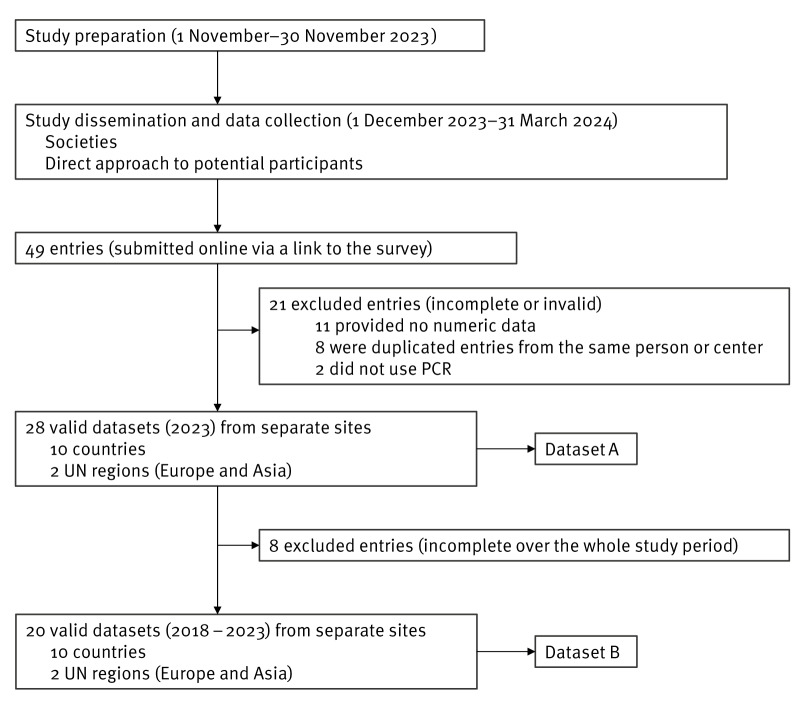
Study dissemination and data collection leading to two complete datasets, encompassing sites in Europe and Taiwan, 1 November 2023–31 March 2024

Following the dissemination of the study, 49 survey answers (entries) were received. Of these, a total of 21 entries were excluded: 11 due to missing numeric data despite reminders, eight were duplicate entries from the same individuals or sites, and two used serology instead of PCR. Two datasets were obtained: Dataset A, comprising 28 sites in 2023, and Dataset B, covering 20 sites from 2018 to 2023 (Figure 1).

UN: United Nations.

Societies: ESCMID: European Society for Clinical Microbiology and Infectious Diseases study groups; ESGMAC: ESCMID study group for Mycoplasma and Chlamydia Infections; ESGMD: ESCMID Study Group for Genomics and Molecular Diagnostics; ESGEM: ESCMID Study Group for Epidemiological Markers; the Swiss Society for Microbiology.

These two datasets were used for further analysis. Most sites were in Europe (n = 27), with one site in Asia (n = 1) (Table 1).

In total, Dataset A (2023 only) included data from 172,223 PCR tests. Among these, 18.7% of the tests were from patients under 18 years old, and 50.0% were from females (more details can be found in Supplementary Table S2). Two and five sites, respectively, did not specify the proportion of children/adolescents under 18 years and females of any age and were therefore excluded from our calculations, resulting in 171,497 tests used to assess PCR detection ratios for patients under 18 years old and 166,564 tests for female patients. Dataset B, covering the period from 2018 to 2023, included a total of 693,106 PCR tests. For sites that provided demographic data in Dataset B (n = 672,558), 14.9% of the tests were for children/adolescents and 50.2% were for females. Overall *C. pneumoniae* detection ratio was 0.31% (± 2 standard deviations (SD):  0.05%) in Dataset A and 0.47% (± 2SD: 0.13%) in Dataset B. Children/adolescents represented 56.4% of positive detections in Dataset A, and 37.1% in Dataset B. Female representation among positive detection was balanced in both datasets (50.0% for A and 52.0% for B).

Of the 28 sites, one site (Denmark) employed a mixed approach, combining in-house and commercial PCR methods. Denmark was considered a site although it provided data from the Danish Microbiological Database (MiBa) [29], which aggregated PCR test results from 10 different microbiological laboratories, all using commercially available PCR-based platforms (Table 1). Denmark was the only site providing country-wide data. Of the other 27 testing sites, seven exclusively used in-house PCR tests, while 19 relied on various commercially available PCR-based platforms.

**Table 1:** Laboratory type, location, method and specific *Chlamydia pneumoniae* PCR technique or manufacturer used and dataset attribution for the study, 27 European sites and Taiwan, 1 November 2023–31 March 2024

Entry number	Laboratory type	City	Country	Methods	Technique or manufacturer	Dataset
1	Hospital or clinical laboratory	Antwerp	Belgium	Singleplex PCR	In-house [31]	A + B
2	Hospital or clinical laboratory	Prague	Czechia	Multiplex PCR	Atypical Pneumonia 8-well (AusDiagnostics)	A + B
3	National data (MiBa)	Copenhagen	Denmark	PCR	Various commercial assays	A + B
4	Hospital or clinical laboratory	Helsinki	Finland	Multiplex PCR	In-house	A + B
5	Hospital or clinical laboratory	Marseille	France	Multiplex and single qPCR	Filmarray Respiratory Panel 2.1 plus (bioMérieux), Fast Track Diagnosis respiratory pathogene (Siemens healthineers), in-house qPCR	A + B
6	Hospital or clinical laboratory	Bordeaux	France	Singleplex PCR	In-house	A + B
7	Hospital or clinical laboratory	's-Hertogenbosch	Netherlands	PCR	In-house	A + B
8	Hospital or clinical laboratory	Sittard-Geleen	Netherlands	Multiplex PCR	In-house	A
9	Hospital or clinical laboratory	Rotterdam	Netherlands	Multiplex PCR	Allplex PneumoBacter Assay (Seegene)	A
10	Hospital or clinical laboratory	Arnhem	Netherlands	Multiplex PCR	Filmarray Respiratory Panel 2.1 plus (bioMérieux)	A
11	Hospital or clinical laboratory	Ljubljana	Slovenia	Multiplex PCR	Chla/Myco pneumo R-GENE (bioMérieux)	A + B
12	Hospital or clinical laboratory	Bilbao	Spain	Multiplex PCR	Allplex Respiratory Panel 4 (Seegene)	A
13	Hospital or clinical laboratory	Barcelona	Spain	Multiplex PCR	Filmarray Respiratory Panel 2.1 plus (bioMérieux)	A + B
14	Hospital or clinical laboratory	Santa Cruz de Tenerife	Spain	Multiplex PCR	Allplex Respiratory panel 4 (Seegene)	A + B
15	Hospital or clinical laboratory	Buchs	Switzerland	Multiplex PCR	Allplex Respiratory Panel 4 (Seegene) and Filmarray Respiratory Panel 2.1 plus (bioMérieux)	A + B
16	Hospital or clinical laboratory	Niederwangen	Switzerland	Singleplex PCR	In-house [17]	A + B
17	Hospital or clinical laboratory	Zurich	Switzerland	Multiplex PCR	Filmarray Respiratory Panel 2.1 plus (bioMérieux)	A
18	Hospital or clinical laboratory	Chur	Switzerland	Multiplex PCR	Filmarray Respiratory Panel 2.1 plus (bioMérieux)	A
19	Hospital or clinical laboratory	Lausanne	Switzerland	In-house duplex PCR	In-house [19]	A + B
20	Hospital or clinical laboratory	La Chaux-de-Fonds	Switzerland	Multiplex PCR	QIASTAT (QIAGEN), Filmarray Respiratory Panel 2.1 plus (bioMérieux), RIDAGENE CAP Bac (r-biopharm)	A + B
21	Hospital or clinical laboratory	Lucerne	Switzerland	Multiplex PCR	Filmarray Respiratory Panel 2.1 plus (bioMérieux)	A + B
22	Hospital or clinical laboratory	Basel	Switzerland	Multiplex PCR	Filmarray Respiratory Panel 2.1 plus (bioMérieux) and Allplex PneumoBacter Assay (Seegene)	A
23	Hospital or clinical laboratory	Lausanne	Switzerland	Multiplex and single PCR	Filmarray Respiratory Panel 2.1 plus (bioMérieux) and CHP/ISEX/025 (Geneproof)	A
24	Hospital or clinical laboratory	Bioggio	Switzerland	Multiplex and single PCR	Filmarray Respiratory Panel 2.1 plus (bioMérieux) and CHP/ISEX/025 (Geneproof)	A + B
25	Hospital or clinical laboratory	Lucerne	Switzerland	Multiplex and single PCR	Filmarray Respiratory Panel 2.1 plus (bioMérieux) and CHP/ISEX/025 (Geneproof)	A + B
26	Hospital or clinical laboratory	Zurich	Switzerland	Multiplex PCR	Respiratory multiplex Lightmix Kit (Roche)	A + B
27	Hospital or clinical laboratory	Geneva	Switzerland	Duplex real-time PCR	BD-MAX AP-2 (Becton Dickinson)	A + B
28	Hospital or clinical laboratory	Taichung	Taiwan	Multiplex PCR	QIASTAT (QIAGEN)	A + B

MiBa: Danish Microbiological Database.

### Significant decrease in cases during the pandemic period

**Figure 2:**
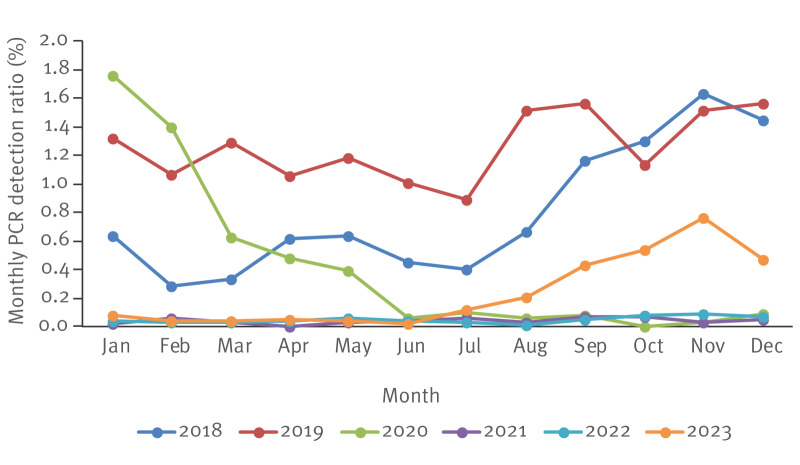
Monthly variation in PCR detection ratios of *Chlamydia pneumoniae*, 19 European sites and Taiwan, 2018–2023

**Figure 3:**
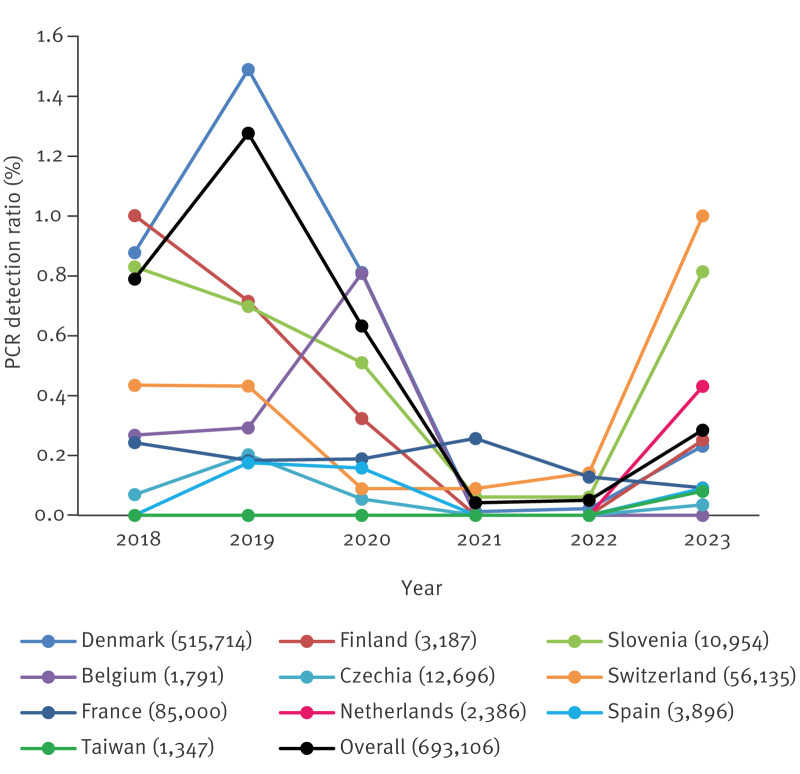
Annual PCR detection ratios for *Chlamydia pneumoniae*, 19 European sites and Taiwan, 2018–2023

An overall decrease in detection ratios was seen between the pre-pandemic and pandemic periods (Figure 2, Figure 3).

Dataset B was used to generate this graph.

Dataset B was used to generate this graph. Denmark was the only country providing country-wide data. Numbers between parentheses are the total number of tests performed.

From an overall detection ratio of 1.05% (± 2SD: 0.22%) during the pre-pandemic period, this dropped significantly to 0.23% (± 2SD: 0.04%) during the pandemic period (p ≤ 0.001). However, some sites continued to see the circulation of *C. pneumoniae*, particularly in France (Marseille), Belgium (Antwerp) and in two sites in Switzerland (Geneva and Niederwangen) (Table 2). At the site level, a significant decrease in detection ratios was only seen in Denmark, Finland, Slovenia and Switzerland (4 of 9 sites), although detection ratios decreased without reaching statistical significance or stayed at 0% in all the other sites except the Belgian site (Table 2, Figure 3).

**Table 2:** PCR detection ratios for *Chlamydia pneumoniae* and detailed numbers of samples over the three study periods for Dataset B, 19 European sites and Taiwan, 2018–2023

Country	City	PCR detection ratios (%)			Number of samples (2018–2019)		Number of samples (2020–2022)		Number of samples (2023)		p value		
		Pre-pandemic period 2018–2019	Pandemic period 2020–2022	Post-pandemic period 2023	Positive	Negative	Positive	Negative	Positive	Negative	2018–2019 vs 2020–2022	2018–2019 vs 2023	2020–2022 vs 2023
Belgium	Antwerp	0.28	0.31	0.00	2	713	3	959	0	114	1.00	1.00	1.00
Czechia	Prague	0.15	0.02	0.04	5	3,417	1	6,453	1	2,819	0.02	0.23	0.52
Denmark	Copenhagen	1.20	0.26	0.23	1,898	155,757	643	246,310	257	110,849	**< 0.01**	**< 0.01**	0.11
Finland	Helsinki	0.85	0.13	0.25	11	1,287	2	1,489	1	397	**< 0.01**	0.32	0.51
France	Marseille	0.23	0.18	0.09	22	9,465	86	46,724	23	25,424	0.31	**< 0.01**	**< 0.01**
France	Bordeaux	0.00	0.00	0.13	0	913	0	1,591	1	751	1.00	0.45	0.32
Netherlands	s-Hertogenbosch	0.00	0.00	0.43	0	491	0	1,199	3	693	1.00	0.27	0.05
Slovenia	Ljubljana	0.75	0.22	0.81	27	3,567	11	5,015	19	2,315	**< 0.01**	0.88	**< 0.01**
Spain	Barcelona	0.00	0.00	1.22	0	101	0	266	1	81	1.00	0.45	0.24
Spain	Santa Cruz de Tenerife	0.13	0.06	0.00	1	758	1	1,660	0	1,027	0.53	0.43	1.00
Switzerland	Lucerne	3.19	0.00	0.48	3	91	0	358	1	206	**< 0.01**	0.10	0.37
Switzerland	Buchs	0.39	0.04	1.46	12	3,088	2	5,513	51	3,440	**< 0.01**	**< 0.01**	**< 0.01**
Switzerland	Niederwangen	0.90	0.62	3.25	2	221	2	321	10	298	1.00	0.08	0.02
Switzerland	Lausanne	0.40	0.00	1.95	7	1,730	0	2,880	29	1,456	**< 0.01**	**< 0.01**	**< 0.01**
Switzerland	La Chaux-de-Fonds	0.66	0.21	0.66	6	897	1	476	21	3,152	0.43	1.00	0.35
Switzerland	Lucerne	0.15	0.00	0.84	3	1,958	0	3,878	16	1,894	0.04	**< 0.01**	**< 0.01**
Switzerland	Bioggio	0.86	0.04	0.71	11	1,262	1	2,597	5	695	**< 0.01**	0.80	**< 0.01**
Switzerland	Zurich	0.04	0.00	0.52	1	2,375	0	5,519	9	1,708	0.30	**< 0.01**	**< 0.01**
Switzerland	Geneva	0.61	0.50	0.45	19	3,088	23	4,598	10	2,191	0.53	0.57	1.00
Taiwan	Taichung	0.00	0.00	0.08	0	0	0	109	1	1,237	1.00	1.00	1.00

Fisher’s exact Test with correction for multiple testing (Benjamini–Hochberg procedure) was used to compare the three periods.

p values in bold indicate statistical significance (p < 0.05).

Four sites located in Taiwan (Taichung), France (Bordeaux), the Netherlands (s-Hertogenbosch) and Spain (Barcelona) reported no cases of *C. pneumoniae* in the pre-pandemic and pandemic periods, indicating a likely complete absence of *C. pneumoniae* circulating in the regions where the sites are located. When looking at the monthly detection ratios between 2018 and 2023 (Figure 2), overall ratios were close to 0% between April 2020 and June 2023, with a mean monthly positivity ratio of 0.064% (± 2SD: 0.004%). This decline in detection ratios coincides with the deployment of COVID-19 prevention measures (lockdowns, school closures) (lockdown periods can be found in Supplementary Table S1 and visualized in Supplementary Figure S1).

### Epidemiology in the post-pandemic period, 2023

**Figure 4:**
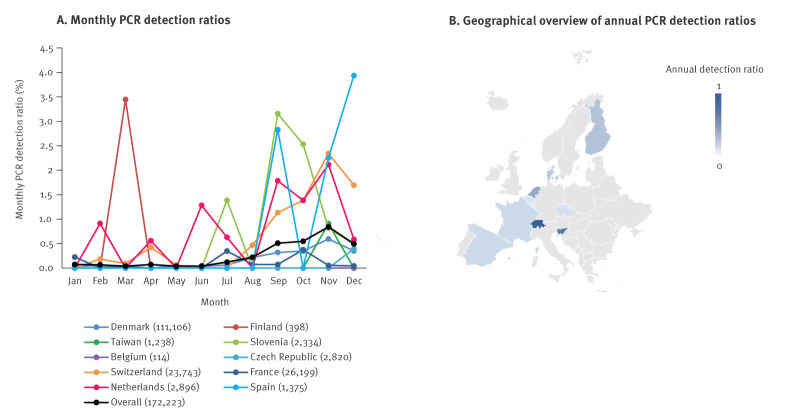
PCR detection ratios for *Chlamydia pneumoniae*, 27 European sites and Taiwan, 2023

The second half of 2023 was marked by an increase in the overall *C. pneumoniae* detection ratios (Figure 2), with important variations between countries (Figure 4A and Figure 4B).

Dataset A was used to generate these figures (raw data and detection rates are available in Supplementary Table S2). Total number of tests are indicated between parentheses. The geographical representation was generated using Bing (Geonames, Microsoft, Open places, OpenStreetMap, TomTom). Denmark was the only site providing country-wide data.

This rebound was observed on the overall data and the detection ratios shifted from a mean of 0.23% (± 2SD: 0.04%) in the pandemic period to 0.28% (± 2SD: 0.04%) in the post-pandemic period (p ≤ 0.002) (Figure 3). When looking at the differences between the sites in the pandemic and post-pandemic periods, statistical significance was only reached in two countries, Switzerland (5/9 sites) and Slovenia (1/1 site), where ratios ranged from 0.52% to 3.25% (Table 2). One site in France (Marseille) had a statistically significant decrease in detection ratios in the post-pandemic compared with the pandemic period. Two sites reported no cases in 2023, in Spain (Santa Cruz de Tenerife) and in Belgium (Antwerp). Several countries continued to experience similar *C. pneumoniae* detection ratios during and after the pandemic period, namely Denmark, Finland and one site in Switzerland (Geneva).

### Differences between the pre-pandemic and post-pandemic periods

Globally, detection ratios in the post-pandemic period remained lower than in the pre-pandemic period (Figure 2, Figure 3), although this difference was not statistically significant (p = 0.65). The differences in detection ratios before and after the pandemic period were statistically significant in six sites. In Denmark and in France, these differences corresponded to a decrease in detection ratios, while in Switzerland (4 sites), there was a statistically significant increase in detection ratios between pre-pandemic and post-pandemic periods.

## Discussion

The study reveals significant epidemiological shifts in the detection of *C. pneumoniae* across various sites, primarily in Europe, during and after the COVID-19 pandemic. The implementation of NPIs such as lockdowns, social distancing, use of facial masks and school closures during the pandemic likely contributed to a marked reduction in the transmission of respiratory pathogens, including *C. pneumoniae*. This is evidenced by the overall decrease in *C. pneumoniae* detection ratios observed during the pandemic period, with some sites reporting almost negligible detection ratios during the height of the pandemic. These results align with other studies that have reported a significant decline in other respiratory infections during the same period, likely due to the widespread implementation of public health measures aimed at curbing the spread of severe acute respiratory syndrome coronavirus 2 [1-4]. Since children/adolescents represented only 18.7% and 14.9% in Dataset A and B, respectively, the larger representation of children/adolescents among all the positive tests (56.4% in Dataset A and 37.1% in Dataset B) likely indicates a higher prevalence among children/adolescents.

However, the post-pandemic period, particularly in the second half of 2023, shows a notable rebound in *C. pneumoniae* detection ratios. This resurgence suggests a possible re-emergence of the pathogen as NPIs were relaxed. The increase in detection ratios was statistically significant in several sites, notably in Switzerland and Slovenia, indicating a potential shift in the epidemiological landscape in the post-pandemic period. Interestingly, while most regions with participating sites showed an increase in detection ratios, a site in France (Marseille) exhibited a statistically significant decrease, suggesting regional variability in how the relaxation of NPIs influenced *C. pneumoniae* circulation.

The persistently lower detection ratios in the post-pandemic compared with the pre-pandemic period, for example in Denmark and certain sites in France, may indicate lasting effects of the pandemic on pathogen circulation or possibly reflect changes in public behaviour and healthcare practices that might have persisted beyond the immediate pandemic response. For example, sustained improvements in hygiene practices or continued cautious behaviour in public spaces could contribute to these lower ratios. However, the duration of these effects may vary depending on the pathogen, as both Denmark and France already experienced resurgences of *M. pneumoniae* in 2023 [7,30].

Conversely, the statistically significant increase in detection ratios in several Swiss sites in the post-pandemic period raises questions about the factors contributing to this rebound. Possible explanations could include a combination of factors such as decreased population immunity due to reduced pathogen circulation during the pandemic, changes in healthcare-seeking behaviour or even shifts in diagnostic practices or criteria. The regional differences observed highlight the complexity of the pandemic’s epidemiological impact and underscore the need for ongoing surveillance to monitor the evolution of *C. pneumoniae* epidemiology, and possibly anticipate rebounds in countries that have not experienced rebounds.

Our study is limited in its geographical representation, as 27 sites are in Europe and only one is in Asia, offering insights only into regions with participating sites. We were not able to obtain data outside Europe and Taiwan. In addition, the number of tests performed varied considerably across sites and countries, limiting the representativeness of the data in some regions. While Denmark provided national data, several countries (Belgium, Czechia, Finland, Slovenia and Taiwan) had only a single participating site. Moreover, the variability in detection methods across sites, with some relying on in-house PCR assays while others use commercial methods, introduces an additional layer of complexity in interpreting these findings. The differences in sensitivity and specificity of these assays could influence detection ratios, potentially contributing to the observed regional differences. Detection ratios may also be impacted by local practices and awareness of the primary-care physician about the different clinical presentation of *C. pneumoniae.* In addition, variability may also arise from differing policies, such as molecular testing being conducted primarily for inpatients at some sites, while others may extend this testing to outpatients as well. It is also possible that a significant number of *C. pneumoniae* infections went undetected, as they may cause only asymptomatic or mild illness in a portion of the population. Since this study focused on molecular diagnosis results, further studies combining molecular and serological prevalence data could provide more comprehensive insights.

## Conclusion

This study provides insights into the evolving epidemiology of *C. pneumoniae* in the context of the COVID-19 pandemic. The findings underscore the profound impact of global non-pharmaceutical public health measures on pathogen circulation and highlight the importance of continued surveillance and research to understand the long-term consequences of the pandemic on respiratory pathogens. Further investigation is needed to explore the underlying factors driving the observed regional differences and to assess the potential long-term changes in the epidemiology of *C. pneumoniae* in the post-pandemic world.

## Data Availability

All data are readily available in the manuscript or the Supplementary material.

## Supplementary Data

346000 Supplementary Material

## Supplementary Data

21000 Supplementary Table S2

## References

[1] Huang QS Wood T Jelley L Jennings T Jefferies S Daniells K Impact of the COVID-19 nonpharmaceutical interventions on influenza and other respiratory viral infections in New Zealand. Nat Commun. 2021;12(1):1001. 10.1038/s41467-021-21157-9 33579926 PMC7881137

[2] Meyer Sauteur PM Beeton ML Uldum SA Bossuyt N Vermeulen M Loens K ESGMAC–MyCOVID Study Team . Mycoplasma pneumoniae detections before and during the COVID-19 pandemic: results of a global survey, 2017 to 2021. Euro Surveill. 2022;27(19):2100746. 10.2807/1560-7917.ES.2022.27.19.2100746 35551702 PMC9101966

[3] Baker RE Park SW Yang W Vecchi GA Metcalf CJE Grenfell BT . The impact of COVID-19 nonpharmaceutical interventions on the future dynamics of endemic infections. Proc Natl Acad Sci USA. 2020;117(48):30547-53. 10.1073/pnas.2013182117 33168723 PMC7720203

[4] Oster Y Michael-Gayego A Rivkin M Levinson L Wolf DG Nir-Paz R . Decreased prevalence rate of respiratory pathogens in hospitalized patients during the COVID-19 pandemic: possible role for public health containment measures? Clin Microbiol Infect. 2021;27(5):811-2. 10.1016/j.cmi.2020.12.007 33352303 PMC7833997

[5] Gouveia C Bajanca-Lavado MP Mamede R Araújo Carvalho A Rodrigues F Melo-Cristino J Sustained increase of paediatric invasive Streptococcus pyogenes infections dominated by M1_UK_ and diverse emm12 isolates, Portugal, September 2022 to May 2023. Euro Surveill. 2023;28(36):2300427. 10.2807/1560-7917.ES.2023.28.36.2300427 37676143 PMC10486195

[6] Alcolea-Medina A Snell LB Alder C Charalampous T Williams TGS Tan MKI The ongoing Streptococcus pyogenes (Group A Streptococcus) outbreak in London, United Kingdom, in December 2022: a molecular epidemiology study. Clin Microbiol Infect. 2023;29(7):887-90. 10.1016/j.cmi.2023.03.001 36925107 PMC10769882

[7] Meyer Sauteur PM Beeton ML Pereyre S Bébéar C Gardette M Hénin N Mycoplasma pneumoniae: delayed re-emergence after COVID-19 pandemic restrictions. Lancet Microbe. 2024;5(2):e100-1. 10.1016/S2666-5247(23)00344-0 38008103

[8] Tagini F Puolakkainen M Greub G On Behalf Of The Escmid Study Group For Mycoplasma And Chlamydia Infections Esgmac . From coughs to complications: the story of Chlamydia pneumoniae*.* J Med Microbiol. 2025;74(4):002006. 10.1099/jmm.0.002006 40279169 PMC12050420

[9] Kauppinen MT Saikku P Kujala P Herva E Syrjälä H . Clinical picture of community-acquired Chlamydia pneumoniae pneumonia requiring hospital treatment: a comparison between chlamydial and pneumococcal pneumonia. Thorax. 1996;51(2):185-9. 10.1136/thx.51.2.185 8711653 PMC473034

[10] Conklin L Adjemian J Loo J Mandal S Davis C Parks S Investigation of a Chlamydia pneumoniae outbreak in a Federal correctional facility in Texas. Clin Infect Dis. 2013;57(5):639-47. 10.1093/cid/cit357 23723194 PMC4678872

[11] Ekman MR Grayston JT Visakorpi R Kleemola M Kuo CC Saikku P . An epidemic of infections due to Chlamydia pneumoniae in military conscripts. Clin Infect Dis. 1993;17(3):420-5. 10.1093/clinids/17.3.420 8218684

[12] Augenbraun MH Roblin PM Mandel LJ Hammerschlag MR Schachter J . Chlamydia pneumoniae pneumonia with pleural effusion: diagnosis by culture. Am J Med. 1991;91(4):437-8. 10.1016/0002-9343(91)90165-T 1951390

[13] Grayston JT Campbell LA Kuo CC Mordhorst CH Saikku P Thorn DH A new respiratory tract pathogen: Chlamydia pneumoniae strain TWAR. J Infect Dis. 1990;161(4):618-25. 10.1093/infdis/161.4.618 2181028

[14] Von Hertzen L . Role of persistent infection in the control and severity of asthma: focus on Chlamydia pneumoniae. Eur Respir J. 2002;19(3):546-56. 10.1183/09031936.02.00254402 11936537

[15] Von Hertzen L Vasankari T Liippo K Wahlström E Puolakkainen M . Chlamydia pneumoniae and severity of asthma. Scand J Infect Dis. 2002;34(1):22-7. 10.1080/00365540110077155 11874160

[16] Kohlhoff SA Hammerschlag MR . Treatment of Chlamydial infections: 2014 update. Expert Opin Pharmacother. 2015;16(2):205-12. 10.1517/14656566.2015.999041 25579069

[17] Reischl U Lehn N Simnacher U Marre R Essig A . Rapid and standardized detection of Chlamydia pneumoniae using LightCycler real-time fluorescence PCR. Eur J Clin Microbiol Infect Dis. 2003;22(1):54-7. 10.1007/s10096-002-0858-2 12582746

[18] Tondella MLC Talkington DF Holloway BP Dowell SF Cowley K Soriano-Gabarro M Development and evaluation of real-time PCR-based fluorescence assays for detection of Chlamydia pneumoniae. J Clin Microbiol. 2002;40(2):575-83. 10.1128/JCM.40.2.575-583.2002 11825973 PMC153405

[19] Opota O Brouillet R Greub G Jaton K . Methods for real-time PCR-based diagnosis of Chlamydia pneumoniae, Chlamydia psittaci, and Chlamydia abortus infections in an opened molecular diagnostic platform. Methods Mol Biol. 2017;1616:171-81. 10.1007/978-1-4939-7037-7_11 28600769

[20] Leber AL Lisby JG Hansen G Relich RF Schneider UV Granato P Multicenter evaluation of the QIAstat-Dx respiratory panel for detection of viruses and bacteria in nasopharyngeal swab specimens. J Clin Microbiol. 2020;58(5):e00155-20. 10.1128/JCM.00155-20 32132186 PMC7180242

[21] Murphy CN Fowler R Balada-Llasat JM Carroll A Stone H Akerele O Multicenter evaluation of the BioFire FilmArray pneumonia/pneumonia plus panel for detection and quantification of agents of lower respiratory tract infection. J Clin Microbiol. 2020;58(7):e00128-20. 10.1128/JCM.00128-20 32350043 PMC7315029

[22] Verkooyen RP Willemse D Hiep-van Casteren SC Mousavi Joulandan SA Snijder RJ van den Bosch JM Evaluation of PCR, culture, and serology for diagnosis of Chlamydia pneumoniae respiratory infections. J Clin Microbiol. 1998;36(8):2301-7. 10.1128/JCM.36.8.2301-2307.1998 9666010 PMC105036

[23] Dowell SF Peeling RW Boman J Carlone GM Fields BS Guarner J Standardizing Chlamydia pneumoniae assays: recommendations from the Centers for Disease Control and Prevention (USA) and the Laboratory Centre for Disease Control (Canada). Clin Infect Dis. 2001;33(4):492-503. 10.1086/322632 11462186

[24] Tuuminen T Palomäki P Paavonen J . The use of serologic tests for the diagnosis of chlamydial infections. J Microbiol Methods. 2000;42(3):265-79. 10.1016/S0167-7012(00)00209-8 11044570

[25] Han HY Moon JU Rhim JW Kang HM Lee SJ Yang EA . Surge of Chlamydia pneumoniae pneumonia in children hospitalized with community-acquired pneumonia at a single center in Korea in 2016. J Infect Chemother. 2023;29(5):453-7. 10.1016/j.jiac.2023.01.012 36738859

[26] Tagini F Opota O Greub G . Chlamydia pneumoniae upsurge at tertiary hospital, Lausanne, Switzerland. Emerg Infect Dis. 2024;30(4):810-2. 10.3201/eid3004.231610 38413241 PMC10977832

[27] R Core Team. R: A Language and Environment for Statistical Computing. Vienna: R foundation for Statistical Computing; 2021. Available from: https://www.R-project.org

[28] Benjamini Y, Hochberg Y. Controlling the false discovery rate: a practical and powerful approach to multiple testing. J R Stat Soc Series B.1995;57(1):289–300.

[29] Voldstedlund M Haarh M Mølbak K MiBa Board of Representatives . The Danish Microbiology Database (MiBa) 2010 to 2013. Euro Surveill. 2014;19(1):20667. 10.2807/1560-7917.ES2014.19.1.20667 24434175

[30] Nordholm AC Søborg B Jokelainen P Lauenborg Møller K Flink Sørensen L Grove Krause T Mycoplasma pneumoniae epidemic in Denmark, October to December, 2023. Euro Surveill. 2024;29(2):2300707. 10.2807/1560-7917.ES.2024.29.2.2300707 38214084 PMC10785206

[31] Loens K Beck T Ursi D Overdijk M Sillekens P Goossens H Evaluation of different nucleic acid amplification techniques for the detection of M. pneumoniae, C. pneumoniae and Legionella spp. in respiratory specimens from patients with community-acquired pneumonia. J Microbiol Methods. 2008;73(3):257-62. 10.1016/j.mimet.2008.02.010 18378345

