# Supplementary material for: Epidemiological changes in Chlamydia pneumoniae molecular detections before, during and after the COVID-19 pandemic in 27 European sites and Taiwan, 2018 to 2023
Source: Euro Surveill. 2025 Jun 12;30(23):2400682. doi: 10.2807/1560-7917.ES.2025.30.23.2400682 (PMC12164279; doi:10.2807/1560-7917.ES.2025.30.23.2400682)
Supplement: Supplementary Material [file 24-00682_GREUB_Supplementary_material.pdf]

This supplementary material is hosted by *Eurosurveillance* as supporting information alongside the article' Epidemiological changes in *Chlamydia pneumoniae* molecular detections before, during and after the COVID-19 pandemic in 27 European sites and Taiwan, 2018 to 2023', on behalf of the authors, who remain responsible for the accuracy and appropriateness of the content. The same standards for ethics, copyright, attributions and permissions as for the article apply. Supplements are not edited by *Eurosurveillance* and the journal is not responsible for the maintenance of any links or email addresses provided therein.

Supplementary materials

Items surveyed in this study

1. Last name
2. First name
3. Academic title
4. Affiliation
5. E-mail
6. Consent to publish data for the ESGMAC ?
7. Consent to be listed as survey participant with name and affiliation in the acknowledgements section of the publication ?
8. Ethical approval for data submission required according to local ethical guidelines ?
9. If yes, please add ethics committee and project number (if available).
10. Type of laboratory / system
11. Name of laboratory / system
12. Link (URL) to the laboratory / system
13. If hospital / clinical laboratory, please specify the type of affiliated hospital
14. City
15. Post / ZIP code
16. Country
17. Comments
18. Technique (e.g. multiplex PCR)
19. Method (e.g. microarray)
20. Product (e.g. Biofire Filmarray Respiratory Panel 2.1)
21. Company (e.g. Biomérieux)
22. Litterature reference (if available)
23. 2023:
    a. Positive test (overall)
    b. Total test (overall)
    c. Positive tests (only <18 year old)

    d. Total tests (only <18 year old)
    e. Positive tests (only female)
    f. Total tests (only female)
    g. Positive tests per month
        i. Jan Feb Mar Apr May Jun Jul Aug Sep Oct Nov Dec
    h. Total tests per month
        i. Jan Feb Mar Apr May Jun Jul Aug Sep Oct Nov Dec

24. 2022:
    a. Positive test (overall)
    b. Total test (overall)
    c. Positive tests (only <18 year old)
    d. Total tests (only <18 year old)
    e. Positive tests (only female)
    f. Total tests (only female)
    g. Positive tests per month
        i. Jan Feb Mar Apr May Jun Jul Aug Sep Oct Nov Dec
    h. Total tests per month
        i. Jan Feb Mar Apr May Jun Jul Aug Sep Oct Nov Dec
25. 2021:
    a. Positive test (overall)
    b. Total test (overall)
    c. Positive tests (only <18 year old)
    d. Total tests (only <18 year old)
    e. Positive tests (only female)
    f. Total tests (only female)
    g. Positive tests per month
        i. Jan Feb Mar Apr May Jun Jul Aug Sep Oct Nov Dec
    h. Total tests per month
        i. Jan Feb Mar Apr May Jun Jul Aug Sep Oct Nov Dec
26. 2020:
    a. Positive test (overall)
    b. Total test (overall)
    c. Positive tests (only <18 year old)
    d. Total tests (only <18 year old)
    e. Positive tests (only female)
    f. Total tests (only female)
    g. Positive tests per month
        i. Jan Feb Mar Apr May Jun Jul Aug Sep Oct Nov Dec
    h. Total tests per month
        i. Jan Feb Mar Apr May Jun Jul Aug Sep Oct Nov Dec

27. 2019:
    a. Positive test (overall)
    b. Total test (overall)
    c. Positive tests (only <18 year old)
    d. Total tests (only <18 year old)
    e. Positive tests (only female)
    f. Total tests (only female)
    g. Positive tests per month
        i. Jan Feb Mar Apr May Jun Jul Aug Sep Oct Nov Dec
    h. Total tests per month
        i. Jan Feb Mar Apr May Jun Jul Aug Sep Oct Nov Dec
28. 2018:
    a. Positive test (overall)
    b. Total test (overall)
    c. Positive tests (only <18 year old)
    d. Total tests (only <18 year old)
    e. Positive tests (only female)
    f. Total tests (only female)
    g. Positive tests per month
        i. Jan Feb Mar Apr May Jun Jul Aug Sep Oct Nov Dec
    h. Total tests per month
        i. Jan Feb Mar Apr May Jun Jul Aug Sep Oct Nov Dec
29. If available, number of tests in 2017
    a. Positive test (overall)
    b. Total test (overall)
    c. Positive tests (only <18 year old)
    d. Total tests (only <18 year old)
    e. Positive tests (only female)
    f. Total tests (only female)
30. If available, number of tests in 2016
    a. Positive test (overall)
    b. Total test (overall)
    c. Positive tests (only <18 year old)
    d. Total tests (only <18 year old)
    e. Positive tests (only female)
    f. Total tests (only female)
31. If available, number of tests in 2015
    a. Positive test (overall)
    b. Total test (overall)
    c. Positive tests (only <18 year old)
    d. Total tests (only <18 year old)
    e. Positive tests (only female)
    f. Total tests (only female)
32. If available, number of tests in 2014

a. Positive test (overall)
b. Total test (overall)
c. Positive tests (only <18 year old)
d. Total tests (only <18 year old)
e. Positive tests (only female)
f. Total tests (only female)

Table S1 – Lockdown and school closure durations per countries

Country	Lockdown duration (days, period)	School closure of primary schools (secondary schools)	Data source
Belgium	52 days (18 Mar–9 May 2020)	114 (182) days	*a*
Czech Republic	51 days (16 Mar-24 Apr 2020; 22 Oct- 03 Nov 2020)	205 (153) days	*a*
Denmark	185 days (11 Mar–17 May 2020; 16 Dec–12 Apr 2021; 17 Dec - 18 Dec 2021)	78 (156) days	*b*
Finland	56 days (18 Mar–31 May 2020)	56 (56) days	*b*
France	102 days (17 Mar–11 May 2020; 29 Oct–14 Dec 2020)	20 (191) days	*a*
Slovenia	87 days (14 Mar–28 Apr 2020; 15 Feb - 19 Mar 2021; 1 Apr -11 Apr 2021)	187 (215) days	*b*
Spain	40 days (14 Mar - 23 Apr 2020)	168 (168) days	*a*
Switzerland	83 days (17 Mar - 27 Apr 2020; 18 Jan - 01 Mar 2021)	59 (59) days	*c,d*
Taiwan	No national lockdown	No school closure	*c*
The Netherlands	185 days (18 Mar - 10 May 2020; 15 Dec 2020 - 22 Jan 2021; 29 Apr - 04 Jun 2021; 28 Nov 2021 - 25 Jan 2022)	128 (171) days	*a*

a, Data extracted from ECDC data (Variable = StayHomeOrder) https://www.ecdc.europa.eu/en/publications-data/download-data-response-measures-covid-19 [accessed on 18.08.2024]; *b*, Data extracted from ECDC data (Variable = Clospubany) https://www.ecdc.europa.eu/en/publications-data/download-data-response-measures-covid-19 [accessed on 18.08.2024]; *c,* Data extracted from https://en.wikipedia.org/wiki/COVID-19_lockdowns#cite_note-495 [accessed on 18.08.2024]; *d,* School closure data were extracted from https://www.swissinfo.ch/eng/society/how-did-covid-19-impact-swiss-education/46018790 [Accessed on 18.08.24]

Figure S1

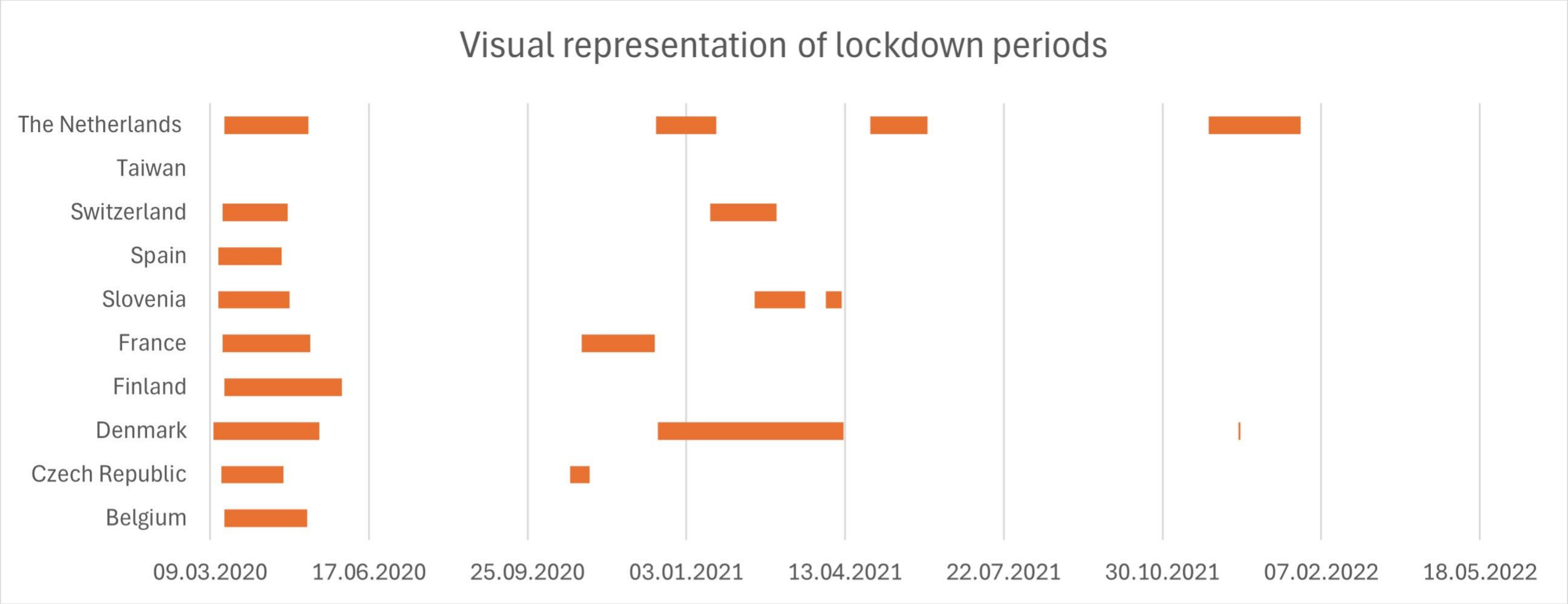
Visual representation of lockdown periods
The Netherlands
Taiwan
Switzerland
Spain
Slovenia
France
Finland
Denmark
Czech Republic
Belgium
09.03.2020
17.06.2020
25.09.2020
03.01.2021
13.04.2021
22.07.2021
30.10.2021
07.02.2022
18.05.2022
